# The early life growth of head circumference, weight, and height in infants with autism spectrum disorders: a systematic review

**DOI:** 10.1186/s12887-023-04445-9

**Published:** 2023-12-08

**Authors:** Roghayeh Molani-Gol, Mohammad Alizadeh, Sorayya Kheirouri, Fatemeh Hamedi-Kalajahi

**Affiliations:** 1 Student Research Committee, Tabriz University of Medical Sciences, Tabriz, Iran; 2 Department of Nutrition, Faculty of Nutrition and Food Sciences, Tabriz University of Medical Sciences, Attar Nishabouri St, 14711, Tabriz, 5166614711 Iran

**Keywords:** Head circumference, Weight, Height, Infants, Autism

## Abstract

**Backgrounds:**

The Autism spectrum disorder (ASD) prevalence has increased significantly over the past two decades. This review summarizes the current knowledge of the association between the early life growth of head circumference (HC), weight, and height with ASD in infants.

**Methods:**

PubMed, Scopus, Science Direct, and Google Scholar databases were searched up to November 2021 using relevant keywords. All original articles are written in English evaluating the early life growth of HC, weight, and height in infants with ASD were eligible for the present review.

**Results:**

Totally, 23 articles involving 4959 infants were included in this review. Of 13 studies that evaluated HC of infants at birth, 10 studies (83.33%) showed that the HC at the birth of autistic children was similar to that of the average found in the control group. Among 21 studies that evaluated the HC and weight status in infants, 19 studies (90.47%) showed that autistic children had larger HC and weight than the control group or abnormal acceleration of head growth during infancy. Height growth of infants was investigated in 13 studies, of which 10 cases (76.92%) reported that infants with ASD were significantly longer than control groups. Most of he included studies had a good quality.

**Conclusions:**

The findings suggest that in infants with ASD, without the contribution of birth growth factors and sex of the child, the growth of HC, weight, and height probably was faster than in infants with normal development, in early life. Therefore, these measurements might be useful as initial predictive biomarkers for the risk of developing ASD.

**Supplementary Information:**

The online version contains supplementary material available at 10.1186/s12887-023-04445-9.

## Introduction

Autism spectrum disorder (ASD) is a neurodevelopmental condition influencing one in 59 children in the world. Along with behavior problems, ASD is accompanied with sleep complications, seizures, gastrointestinal difficulties, and mental health concerns that can intensely affect the quality of life of children and their families [1]. Intellectual disability or low verbal ability were also reported in around one-third of children with ASD [1]. Timely identification of developmental disorders is vital to the well-being of children. American Academy of Pediatrics (AAP) suggests that developmental surveillance and standardized screening tests be considered for ASD at 18 and 24 months of age, at every health supervision visit [2].

The precise cause of ASD is mostly unknown [3]. According to evidence, multiple risk factors including genetic, environmental, immunological, and perinatal factors may contribute to the ASD pathogenesis [3–6]. On the other hand, both genetics and environmental factors early in development have role in the etiology of autism [7]. However, some of the evidence supporting a significant contribution of environmental factors to autism risk [6]. Features of autism may be detected in early childhood, but the diagnosis of autism is usually not made until much later [5]. Recently, ASD detection strategies focus on early risk markers and growth abnormalities during the early years of life have often been paid attention, to in this direction [8–10].

Growth assessments and standard growth patterns are the gold standard diagnostic tests by which clinicians evaluate the health and physical and emotional well-being of a child. Height, weight, and head circumference (HC) are the main components of anthropometry for infants and toddlers less than two years of age [11]. Reliable sequential anthropometric assessments can help recognize underlying medical, nutritional, or social problems in infants and children [12].

Poor growth during infancy or childhood may contribute to adverse health outcomes in adults [13, 14], hence prevention of growth retardation at these periods of life may have both short- and long-term health advantages [15, 16]. On contrary, several observational investigations have indicated that early life (under two years) accelerated or too fast growth may adversely contribute to long-term health outcomes such as increasing the risk of obesity and chronic diseases [17–19]. In preterm infants, the growth acceleration may be beneficial for later neurodevelopment [17], but it may affect contradictory in healthy term infants. Recently, infancy growth accelerated has become a major focus of research.

Increased body growth, including weight and height, during infancy, has been suggested as a feature of ASD by several studies [9, 20]. In addition, macrocephaly and abnormal acceleration in head growth during the early stages of postnatal development have been proposed as a discerning feature of ASD, by some other investigations [21, 22]. However, the findings were inconsistent across the studies and it is not yet definitively known whether accelerated HC and body growth during infancy are associated with ASD incidents. Moreover, most studies have focused on the physical growth in older children and adolescents with ASD. Best to our knowledge, there is no comprehensive study to review systematically the association between early life growth of HC, weight, and height with ASD incidents in infants. Therefore, the present study was performed to summarize the existing literature regarding the association between early life growth of HC, weight, and height with ASD incidents in infants.

## Methods

The protocol of this study was approved and registered by the Research Vice-Chancellor of Tabriz University of Medical Sciences (ethical code: IR.TBZMED.REC.1400.1243).

### Search strategy

This systematic review was conducted according to the Preferred Reporting Items for Systematic Reviews and Meta-Analyses (PRISMA) guidelines (Supplementary Table 1) [23]. Electronic databases of PubMed, ScienceDirect, Scopus, and Google Scholar were searched without date restrictions until November 2021, using the following keywords: “weight OR height OR length OR head circumference OR growth OR nutritional status” AND “early life OR first year of life OR early growth” AND “autism spectrum disorders OR ASD OR autism OR autistic” AND “children OR infants OR infancy”. We defined the early life as the first 2 years of life. Moreover, to ensure the inclusion of all eligible studies, a separate search was conducted through Google, and also the reference lists of the included studies were reviewed. The search strategy is shown in Supplementary Table 2.

### Articles screening and selection criteria

The extracted studies were saved in an EndNote software and duplicate studies were removed. To identify studies with the correct scope for the current review, the remaining articles were screened in two stages by two independent reviewers (SK and RMG). At first, the titles and abstracts of all the articles were checked. Then, full-text of the remained articles was scrutinized to ensure the suitability of the study for inclusion in this review. Finally, original English-language articles that meted the eligibility criteria were selected. All clinical and observational studies that addressed the early life (ages < 2 years) growth of HC, weight, and height in infants with ASD were eligible for inclusion in this study. Reviews, abstracts, conference papers, editorials, book chapters, posters, letters, thesis, animal, and generic studies were not included. Studies that measured the association between prenatal head growth that was estimated by sonography and postnatal autistic traits were excluded. Studies that measured the association of growth factors and autistic traits in ages > 2 years were excluded. Articles that studied other autistic traits (e.g., oral motor coordination and muscle tone) in early life (case study) and used patients with two or more neurodevelopmental disorders were also excluded. Then, the full texts of the screened articles were critically and separately analyzed for eligibility.

### Data extraction

The extracted data from the eligible studies were following: the authors’ name; year of publication; study design and location; the number of participants; mean age and gender of participants; mean HC, weight, and height at birth; percent of macrocephaly; findings concerning the early life growth of HC, weight, and height in infants with ASD.

### Articles quality assessment

The assessment of each included study quality was done using the Newcastle-Ottawa scales [24]. Assessment is based on three sections of the comparability, selection, and the exposure or outcome of the groups. The adopted Newcastle-Ottawa scale score for cross-sectional studies is maximum of 10 points and if the overall score was within 7 to 10 points, the study has good quality [24, 25]. The Newcastle-Ottawa scale score is maximum of 9 points for case-control studies and if the overall score was within 6 to 9 points, the study has good quality [24, 26].

## Results

### Study selection

**Fig. 1:**
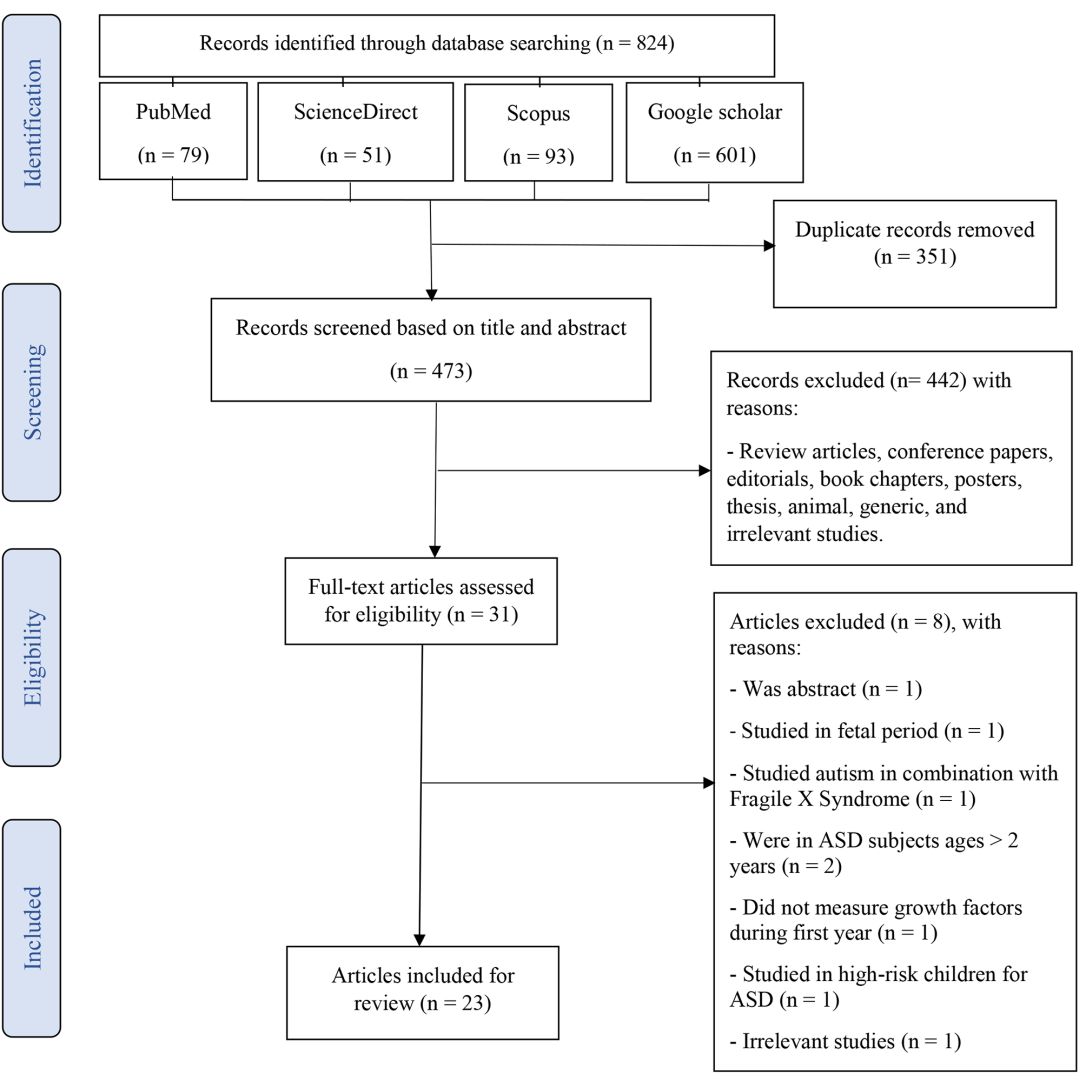
PRISMA diagram for the process of the search and study selection

The process of search and study selection (PRISMA diagram) of this systematic review is showed in Fig. 1. Totally, 504 potential articles were retrieved through searching of PubMed (n = 79), Google Scholar (n = 601), Scopus (n = 93) and ScienceDirect (n = 51) databases. Following the elimination of duplicate articles, 473 studies remained for additional screening. Of these, 442 studies were excluded in the first stage of screening based on the title and abstract of the articles. During critical analysis, 31 articles were screened of which 8 articles were excluded because of non-availability of full text (n = 1), being out of the studied life stage (fetal period) (n = 1) [27], studying autism in combination with Fragile X Syndrome (n = 1) [28], were at ages > 2 years (n = 2) [29, 30], did not measure growth factors during the first year (n = 1) [31], were on high-risk children for ASD (siblings of a child with ASD) (n = 1) [32], and were unrelated (n = 1). Finally, 23 articles were included in the current systematic review.

### Quality of articles

As presented in Supplementary Tables 3 and 4, the quality of each included study was assessed using the Newcastle-Ottawa scales for cross-sectional and case-control studies resulting in the mean scores of 7.6 and 7.5, respectively (Supplementary Tables 3 and 4). These findings indicated that the included studies had a good quality. The most of studies had enough sample sizes and represented infants of the wider community thus scoring well in the selection domain. Scores about the comparability domain were relatively good due to all of the reviewed studies did not adjust all potentially important confounders.

### Characterizes of the included studies

The main characteristics of the included studies are shown in Table 1. Of 23 included studies, 16 studies had a retrospective design that used medical records, and 7 studies were conducted prospectively. Both genders had been included in 19 studies, but in 4 cases, it had only been restricted to males. A total of 4959 autistic children were included in this study.

**Table 1:** Characteristics of the included studies

Citation (First author et al. year)	Study design/ Country	Nomber of participants	Gender	Mean age (year)	Macrocephaly rate	HC, weight, and height at birth	Growth of HC	Growth of body weight	Growth of body height	Confounders considered in the analysis	Outcomes
Chawarska K/ 2011 [37]	Retrospective/ Atlanta/Georgia	Autistic: 64 Normal: 55	Male	Autism: 2.2 ± 0.05 Normal: 2.0 ± 0.7	15–18 months: ASD 13.6% Control 11.8%	Boys in autism and control groups had similar HC, weight, and height shortly after birth.	Autistic boys had significantly larger HC than that of TD controls by 9.5–18 months (p < 0.05).	Autistic boys were significantly weighed more by age 11.4 months and remained significant throughout 12, 15, 18, and 24 months (p < 0.05).	Autistic boys were significantly longer by age 4.8 months. Their height remained significantly above that recorded in TD controls at 6, 9, 12, 15, 18, and 24 months (p < 0.05).	-	Autistic boys experienced generalized overgrowth at 6.5–24 months, from those of TD controls.
Constantino J/ 2009 [80]	Retrospective/USA	Autistic: 48 Control: 85	Male	4–18 years	Not reported	Not reported.	The differences between the HG rates in the ASD and control groups was significant only at birth-6 months (p = 0.04).	-	-	-	Rate of HG of ASD subjects was slightly accelerated compared to controls, but not significant.
Courchesne E/ 2003 [83]	Prospective/ USA	ASD: 48 (2–5 years)	Male/Female	2–5 year	6–14 months: ASD 53% Healthy 6%	In ASD infants, birth HC was smaller compared to healthy infants (p < 0.001).	Within the ASD group, excessive increase in HC was accured between 1–2 months and 6–14 months while, only 6% of healthy infants had accelerated HC growth.	Body weight did not differe from healthy infants at any time points.	Body height did not differe from healthy infants at any time points.	-	ASD infants had a reduced head size at birth and a sudden and excessive increase in head size between 1–2 months and 6–14 months.
Daniel J/ 2014 [8]	Retrospective/USA	ASD: 200 Normal: 147	Male/Female	Birth to 2 years	At birth (p = 0.65): ASD 1.0% TD 2.0% At 24 months (p = 0.47): ASD 6% TD 4.1%	There was no statistically significant difference between boys and girls with ASD and TD controls in HC, weight, and height at birth.	Between 10.7–22.8 months, boys with ASD had significantly larger HC than TD boys (p < 0.05). No statistically significant differences in HC were observed in girls with ASD compared to TD girls at any age.	Autistic boys were significantly heavier than TD boys by 8.7 months, with the difference by 24 months. Girls with autism and PDDNOS showed a similar albeit nonsignificant trend, becoming heavier than TD girls by 0.70 kg by 24 months.	Autistic boys grew significantly longer than TD boys by 3.6 months. Girls with ASD showed a slight increasing trend, becoming on average 0.77 cm taller than TD girls by 24 months, these effects were not statistically significant.	Gestational age	Boys but not girls with ASD were larger and exhibited an increased rate of extreme EGO compared to community controls
Dawson G/ 2007 [82]	Retrospective/USA	ASD: 28	Male	37–52 months	Not reported	Not reported	HC z-scores relative to norms significantly increased in the autism sample from birth to 12 months (p < 0.001), but this pattern did not persist beyond 12 months.	-	Length/height is significantly related to HC; children who are longer/taller tend to have larger heads.	Height	A period of exceptionally rapid HG occurs during the first year of life in autism; after 12 months of age, the rate of HC growth decelerates relative to the rate during the first year of life.
Dementieva Y/ 2004 [21]	Retrospective/USA	ASD: 251	Male/Female	3–21 years Average age 8.15 ± 4.43	Birth to 12 months (p < 0.0001): ASD 19% (66% males and 34% females) The general population 3.0%	Not reported	There as an abnormal acceleration in HG during the first and second months of life in a subgroup of autistic individuals.	-	-	Multiple comparison adjustments-familial similarities	-
Fukumoto A/ 2008 [83]	Retrospective/Japan	ASD: 85	Male/Female	-	Not reported	The HC, weight, and height at birth of autistic children was similar to that of the average found in control group.	HC increased at 1 month after birth. The discrepancy reached a peak at 6 months, while the difference became smaller at 12 months.	Boys from the high functioning group showed significant differences in body weight at 3, 6 and 12 months. Boys from the low functioning group (IQ < 70) showed a similar difference at 3 and 12 months.	The body length was significantly greater than the standard values only in the high functioning group (IQ ≥ 70) of boys at 3 months and 6 months.	-	
Fukumoto A/2011 [84]	Retrospective/Japan	ASD: 280 Controls: 609	Male/Female	ASD: 7.39 ± 3.64 years Controls: 7.02 ± 2.56 years	Not reported	At birth, neither HC nor weight significantly differed between the ASD and control groups among the males and females.	Only the HC in the male ASD group was significantly increased from 6 to 9 months after birth, reaching a peak at 6 months after birth. No difference was found in the female ASD group.	-	-	Height, weight, and age.	
Gillberg Ch/2002 [85]	Retrospective/Sweden	ASD: 50	Male/Female	Mean age: 6 years 4 months	ASD: Birth 35.5% After 16 month 26%	Mean OFCs of ASD at birth were larger than normative values (p < 0.05).	26% of children were macrocephalus after age 16 months.	-	-	-	
Grandgeorge M/2013 [9]	Retrospective/France	ASD: 422 Control: 153	Male/Female	18 months to 18 years	ASD: Birth 5.7% With regard to body length 11.4% With regard to body weight 7.6% Control: Birth 3%	The individuals with ASD did not significantly differ from the control group at birth (p > 0.05).	Relative macrocephaly with regard to body length in ASD was significantly higher than the rate in the control group.	-	-	Height and weight,	
Hazlett H/2005 [86]	Retrospective/USA	ASD: 51 Control: 25	Males/Females	18–35 months	Not reported	No group differences between HC ratings were found at birth.	There as a significantly increased rate of HC growth appearing to begin around 12 months of age.	-	-	Age and sex	-
Kral T/2018 [87]	Retrospective/USA	ASD: 668 Control: 884	Male/Female	59.3 ± 6.6 (2–5 years)	Not reported	Not reported	-	ASD children showed the highest frequency of rapid weight gain (44%) and were 3.47 times more likely to be overweight/obese (p < 0.001).	-	Maternal education, race/ethnicity, poverty status, birth weight, child age, and sex.	-
Marz K/2007 [22]	Retrospective/USA	ASD: 35	Male/female	ASD: 26.6 months ± 4.5 Control: ages 19 months to 10 years	Not reported	Not reported	Infants with ASD had a significantly smaller HC at birth to 2 weeks and a significantly larger HC by 10 to 14 months. However, when overall length and weight were controlled, HC was not bigger in the ASD group compared to local controls.	Children with ASD were also significantly heavier beginning at 1 to 2 months.	Children with ASD were significantly longer beginning at 1 to 2 months.	-	-
McKeague l/2015 [88]	Retrospective/USA	ASD: 468 ASD: 468	Male/Female	5.1 ± 3.4	Not reported	There was not significant difference between birth weight of ASD and control groups.	Growth velocity of HC at 3 months of age, is significantly associated with autism (p = 0.014).	Weight growth velocity at 18 months without ID (p = 0.02) and 24 months without ID (p = 0.042).	Height growth velocity among subjects with autism and without ID is significantly associated with autism at 6 months (p = 0.007).	Maternal age, paternal age, gestational age, birth weight, previous births, and maternal socioeconomic status.	
Mraz K/2009 [89]	Retrospective/USA	ASD: 24 Control: 37	Male/Female	4 years 10 months to 9 years 8 months	Not reported	Not reported	HC growth were significantly greater in ASD groups compared with controls (p < 0.05).	Weight growth were significantly greater in ASD groups compared with controls (p < 0.05).	Mean length in the ASD group did not differ significantly from controls (p < 0.05).	Sex and age.	
Muratori F/2012 [90]	Retrospective/Italy	ASD: 50 Control: 100	Male/Female (40/10)	Mean age: 52 months	6–12 months: ASD 18%	At birth there was no significant difference in HC size between ASD and TD groups.	At 3–12 months HC was significantly greater in ASD group compared to healthy infants, but not at 1–2 months. The HC growth was significantly greater in ASD compared to TD group (p = 0.001)	Weight was significantly smaller in ASD group compared to TD at 1–12 months and no difference was found on the rate of weight growth (p = 0.655) between the two groups.	No significant differences were found between the two groups (p = 0.491) on the rate of height growth.	Weight and height.	-
Rommelse N/2011 [91]	Retrospective/Netherlands	ASD: 129 Non-ASD psychiatric disorders: 59	Male/Female (109/20)	0–20 years	There were no differences between the ASD and control groups. ASD: 1–4 months: 1.2% 5–9 months: 1.6% 10–13 months: 4.7%	Both groups had a decreased height at birth (ASD: p = 0.001; PC: p = 0.02).	ASD children had an increased HC relative to their height at first month (p = 0.001) and 2 months (p = 0.006).	There was a decreased weight very early in Development (first month) in both groups (ASD: p = 0.001; PC: p = 0.005).	Both groups had a decreased height at 1 month of age (ASD: p = 0.001; PC: p = 0.005).	Sex and age.	Neither group showed abnormal acceleration of HC or weight, but both groups showed a significant abnormal acceleration of height between birth and 14–19 months of age.
Schrieken M/2013 [10]	Retrospective/Netherlands	ASD: 96 Control: 163	Male/Female (81.2% boys)	1–3 years	Not reported	ASD children had somewhat smaller HC at birth than control children.	With increasing age, children with ASD had a proportionally smaller HC compared to their height (p = 0.003) the first 13 months of life.	-	-	Height	-
Suren P/2013 [92]	Prospective/United Kingdom	ASD:376	Male/Female (310/66)	3.6–13.1 years (mean 7.4 years)	ASD: Birth-12 months 4.3–8.7%	At birth, mean HC for boys with ASD was close to the mean of HC for boys without ASD.	In boys with ASD, mean HG was similar to that of other boys, but variability was greater. Throughout the first year, the HC of girls with ASD was reduced by 0.5 cm at 12 months.	The mean weight of ASD girls was 150–350 g below at all ages from birth to three years. ASD boys had mean weight 300 g above the control mean at age 12 months.	The mean length of ASD was similar to that of other girls. ASD boys had mean length 1.1 cm above the control mean at age 12 months.	Parental height, parental education, maternal smoking, parity, gestational age at birth, and breastfeeding.	-
Torrey F/2004 [93]	Prospective / Maryland	ASD: 15 Control: 50,000	Male/Female (11/4)	7 years	ASD: Birth 13·3% 6–14 months 6·7%	The autistic group had a slightly but not significantly larger HC at birth.	At 4 months, the HC in the autistic group was not significantly larger than that of control subjects.	Body weight was significantly larger in the autistic group.	Body length was significantly larger in the autistic group.	-	-
Van Daalen E/2007 [94]	Prospective/ Netherlands	ASD: 53 Control: 22	Male/Female (44/9 9/13)	3 years	ASD: 1–12 months 11.3%	Not reported	Growth of HC was normal in children with ASD compared with controls in the first year of life.	-	Growth of body length was accelerated in children with ASD compared with controls in the first year of life.	-	Accelerated growth between 1–6 months.
Webb S/2007 [95]	Prospective/ USA	ASD: 28 With developmental delay without autism: 8	Male	3–4 years	ASD: Birth-36 months 21.4%	Occipitofrontal circumference of ASD did not differ frome normal values.	Compared with the normal values, occipitofrontal circumference for the ASD group was statistically significantly smaller at age 1 to 6.99 months (p < 0.005) but not at birth to age 0.99 months or from age 7 to 36 months.	-	Compared with normal values for height, the ASD group, on average, was statistically significantly longer or taller than the norms only during the first month of life.	Bonferroni adjustment	Rate of growth from birth to age 36 months was statistically significantly higher for the ASD group than the developmental delay group.
Raghavan R/ 2018 [56]	Prospective/ Maryland	ASD: 822	Male/Female	5.2–9.8 years	Not reported	Birthweight was significantly higher in control than in ASD group (p = 0.03).	-	Extremely rapid weight gain during infancy was associated with a greater ASD risk (OR: 3.11; 95% CI: 1.37, 7.07).	-	Maternal age at delivery, smoking during maternal status, child’s sex, gestational age at birth, year of the baby’s birth, and mode of feeding.	-

ASD: Autism spectrum disorder, ID: Intellectual disability, CI: Confidence interval, HC: Hip circumference, HG: Head growth, OR: Odd ratio, TD: Typically developing, PDD-NOS: Pervasive developmental disorder not otherwise specified, POP: Population controls

#### Relationship between early life HC growth with ASD

In all, 21 studies assessed the status of HC growth in ASD infants that of which 13 cases reported information regarding HC in children at birth. 83.33% of the studies (10 of 13) showed that the HC at the birth of autistic children was similar to that of the average found in the control group, two cases indicated smaller HC, and one study reported a larger HC in ASD infants than in control groups. Among 21 studies that evaluated the HC status in infants, 19 studies (90.47%) showed that autistic children had larger HC or abnormal acceleration of HC growth than the control group. In 12 cases of the included studies, the percent of macrocephaly (HC > 97th percentile or HC > 2 standard deviations (SD)) in ASD infants ranged from 1.2 to 53% that these results were consistent with standard macrocephaly rates according to CDC norms and only in two studies were more than standard values. Nineteen studies were conducted on both sexes, of which 14 studies (73.68%) reported similar results between males and females, and three studies showed greater mean HC in males than females.

#### Relationship between early life weight gain with ASD

Six studies reported data about the birth weight of ASD infants, of which five cases showed no significant difference between ASD and typically developed infants. One study revealed that the birth weight of autistic infants was lower than that in the control group. Twenty-one studies assessed the weight status of children during the infancy period, of which 19 cases (90.47%) demonstrated that weight growth was significantly greater in ASD groups compared with controls. Of studies conducted on both genders (n = 19), all of them reported similar findings in males and females, only one case indicated higher weight gain in boys rather than in girls, during infancy.

#### Relationship between early life length growth with ASD

Regarding height at birth, five studies indicated a similar height in ASD and control infants. Thirteen studies investigated the height growth of children during the infancy period, of which 10 cases (76.92%) reported that infants with ASD were significantly longer than control groups. Among studies that included both genders, one case indicated larger height in boys rather than in girls, and the other remaining reported similar results in both sexes.

## Discussion

The findings of the present study showed that rapid growth of weight, height, and HC during infancy was associated with an enhanced risk of ASD developing. The previous research also emphasis the importance of assessing neonatal anthropometric measures such as weight and HC growth [33–35]. Some studies confirm the validity of HC growth as a potential biomarker of normal and abnormal early child development [35, 36]. Recently, it has been reported that an abnormality in factors such as hormone levels, metabolism, and growth or neurotrophic factors may predispose a subject to overall acceleration of growth as well as ASD [22, 36, 37]. Neurotrophins abnormalities, which regulate the metabolism of glucose, may cause accelerated growth in HC and body size [38] and also improve neuronal growth and survival [39]. Liu et al. [40] performed a comprehensive systematic review on the changes in peripheral neurotrophic factors in autistic children and revealed strong evidence that the levels of brain-derived neurotrophic factor (BDNF), nerve growth factor (NGF), and vascular endothelial growth factor (VEGF) in peripheral blood of autistic children were higher than those in healthy controls, establishment the evidence that neurotrophic factors play critical roles in ASD onset and/or development. However, they found no significant associations between neurotrophin-3 (NT-3) or neurotrophin-4 (NT-4) and ASD [40]. It has been reported that overgrowth of the brain volume in early ASD children is largely due to the augmented proliferation of neural progenitor cells [43]. Neurotrophic factors have a positive role in the embryonic neural progenitor cells proliferation [42]. Importantly, the BDNF level is temporally regulated during development that has been shown to be required for the proper development and functions of neuronal [43]. Therefore, it is likely that in the early life of ASD children, abnormal regulation of BDNF cause the subsequent long-term changes in structure and function of the brain, such as accelerating its growth. BDNF has also similar effects in other psychiatric disorders [44].

According to the previous evidence, abnormal secretion of some hormones, such as adiponectin, leptin, ghrelin, insulin, and IGF-1 may cause changes in the growth process, especially in early life, and may be associated with the risk of autism [45–50]. Adipokines, cytokines secreted mostly by adipose tissue, may have a widespread effect and function on brain health [51]. Rodrigues et al. found significant changes in the adipokines plasma levels in ASD patients [52]. Many adipokines such as interleukin-8 [53], interleukin-6 (IL-6) [54], IL-10 [55], leptin [56], tumor necrosis factor-alpha (TNF-α) [57], omentin [58], resistin, and adiponectin [59] have been shown to be associated with ASD. Adiponectin is an adipocyte-derived circulating protein that based on recent evidence may have a role in the pathophysiology of autism [60]. Fujita-Shimizu et al. indicated that the serum levels of adiponectin were significantly lower in the individuals with autism than in normal subjects [61]. However, the role of adiponectin on the early life growth of autistic infants is not clear.

Leptin is another hormone whose abnormality in its release may be contributed to the association between rapid growth and ASD. Recent works found elevated plasma leptin levels in children with autism compared to typically developing controls [52, 62, 63]. There are some reasons for the involvement of leptin in the neuropathology of ASD. Leptin can cross the blood-brain barrier through active transport [64]. Moreover, it has been reported that proinflammatory cytokines can mediate the rapid postnatal growth of ASD [65]. Raghavan et al. indicated the mediating effect of leptin in extremely rapid weight gain during infancy in ASD children [56]. Although they did not analyze HC, weight is the strongest predictor of HC during most of the infancy [22]. In agreement with this, animal models showed that rapid catch-up growth in early childhood is connected with leptin resistance [66]. Beyond leptin’s role in weight gain of postnatal, this pleiotropic cytokine has an essential function in the neurodevelopment regulation of including neural differentiation, promoting migration of neuronal lineage cells to the cortical plate, synaptic plasticity, and neuron excitability, [62, 63, 67].

It is well known that ghrelin has a significant role in the growth and development of infants [68–70]. Al-Zaid et al. showed that the plasma levels of ghrelin were significantly decreased in the autism group compared with healthy controls and revealed also a significant negative association between ghrelin and HC [50]. It is intriguing the fact that autistic children despite low ghrelin levels have increased weight; this could be suggesting a drastic disturbance in the central processing of these peripheral signals. The involvement of many other hormones which may have functions in body weight management is another possible explanation [50].

Insulin-like growth factor-1 (IGF-1) is a neurotrophin that regulates somatic growth and metabolism and myelination and brain growth [71]. Lower plasma IGF-1 and growth hormone-binding protein (GHBP) was observed in autistic children [46], and IGF-1 levels were positively correlated with HC [46]. Li et al. also demonstrated that the ASD group had a significantly lower serum level of IGF- 1 and IGFBP (IGF binding protein)-3 than the control group [72]. The beneficial effects of IGF-1 for treating ASD are due to the potent effects of IGF-1 on synaptic maintenance, function, and plasticity. Based on these findings, the reduction in serum IGF-1 levels in early childhood may be associated with the development of ASD, but the mechanism of the association between decreased IGF-1 levels and increased HC, weight, and height in infants with autism has not yet been elucidated. Abnormal regulation of some hormones such as BDNF, IGF-1, leptin, and ghrelin may be associated with the accelerated growth of infants. However, genes related to head circumference and behavioral development, such as PTEN, might have a role in ASD [73–75]. Fu et al. revealed that some of the PTEN variant and ASD genetic background affected genes involved in neurogenesis, neural development, and synapse signaling and contribute to cellular features consistent with ASD associated with macrocephaly [74]. Moreover, mutations in the regulator genes of mTORC1 result in ASD-like phenotypes through the disruption of the mTORC1-mediated signaling [75].

Macrocephaly is one of the most common physical findings was achieved in autistic individuals [76]. Reported rates of macrocephaly in autistic children vary, with an average rate of 20%. However, 83.3% of the studies included in this review showed that autistic children at or shortly after birth usually did not have macrocephaly and had a similar HC percentile distribution as the general population. Therefore, an abnormal acceleration in head growth during the earliest stages of postnatal development, independent of macrocephaly, may be a risk factor related with the development of ASD [21].

Timely evaluation and early detection of ASD among young children should be an important public health goal [77] because evidence showed that early treatment and services for ASD links with improved outcomes [40, 78, 79]. It should be considered that the findings were obtained from the studies with various follow-up times and different time points for assessing growth and outcomes and were concluded from retrospective and prospective data. Moreover, racial difference between the infants may be have a role in these findings. Nevertheless, early life assessment of anthropometric measures is suggested.

### Limitation of the study

Since growth factors had been measured at various time points of infancy across the studies, we were not able to carry out a meta-analysis. Moreover, the majority of the included studies not reported the criteria that were used for the diagnosis of the ASD infants. The sample sizes of the included studies also were low so, generalizing it to all ASD populations should be done with caution. Some environmental factors such as socioeconomic status of ASD infants’ families may contributed to growth and developmental changes in ASD children. However, this issue has not been considered in the included studies in this review.

## Conclusions

More than 91% of the studies included in this systematic review revealed that rapid growth of HC, weight, or height during infancy probably was associated with the risk of ASD. Therefore, these measurements might be helpful as initial predictive biomarkers for the risk of developing ASD. In addition, these measurements may be a useful and cost-effective method in helping to diagnose the risk of ASD in situations where there isn’t access to magnetic resonance imaging technology. However, these measures are not replaceable with aspects that neuroimaging exams can achieve. Long-term prospective cohort studies are required to determine the association between accelerated growth in the early life and ASD risk.

### Electronic supplementary material

Below is the link to the electronic supplementary material.

Supplementary Material 1

Supplementary Material 2

Supplementary Material 3

Supplementary Material 4

## Data Availability

Data are available from the corresponding author upon reasonable request.
