# Supplementary material for: The early life growth of head circumference, weight, and height in infants with autism spectrum disorders: a systematic review
Source: BMC Pediatr. 2023 Dec 8;23:619. doi: 10.1186/s12887-023-04445-9 (PMC10704616; doi:10.1186/s12887-023-04445-9)
Supplement: Supplementary file 2 — Supplementary Material 2 [file 12887_2023_4445_MOESM2_ESM.docx]

**Supplementary Table 3.** Newcastle-Ottawa scale for assessment the quality of six included cross-sectional studies evaluating the early life growth of head circumference, weight, and height in infants with autism spectrum disorders.

| Study ID | Quality assessment criteria | | | | | | | | | | | | | | | | | | | | Overall Quality Score (Maximum = 10) |
| --- | --- | --- | --- | --- | --- | --- | --- | --- | --- | --- | --- | --- | --- | --- | --- | --- | --- | --- | --- | --- | --- |
|  | **Selection** | | | | | | | | | | | | **Comparability** | | **Outcomes** | | | | | |  |
|  | **Representativeness of the sample** | | | | **Sample size** | | **Non-respondents** | | | **Ascertainment of the exposure** | | |  |  | **Assessment of the outcome** | | | | **Statistical test** | |  |
|  | Truly representative of the average in the target population | Somewhat representative of the average in the target population | Selected group of users | No description of the sampling strategy | Justified and satisfactory | Not justified | Comparability between respondents and non-respondents characteristics is established, and the response rate is satisfactory | The response rate is unsatisfactory, or the comparability between respondents and non-respondents is unsatisfactory | No description of the response rate or the characteristics of the responders and the non-responders | Validated measurement tool | Non-validated measurement tool, but the tool is available or described | No description of the measurement tool | The study controls for the most important factor | The study control for any additional factor | Independent blind assessment | Record linkage | Self-report | No description | The statistical test used to analyze the data is clearly described and appropriate, and the measurement of the association is presented | The statistical test is not appropriate, not described or incomplete |  |
| Courchesne E/ 2003 [66] |  |  |  | NS | * |  | * |  |  | ** |  |  | * | NS | ** |  |  |  | NS |  | 7 |
| Dawson G/ 2007 [67] | * |  |  |  | * |  | * |  |  | ** |  |  | NS | NS |  | ** |  |  | NS |  | 7 |
| Fukumoto A/ 2008 [68] | * |  |  |  | * |  | * |  |  | ** |  |  | * | NS | ** |  |  |  | * |  | 9 |
| Gillberg Ch/2002 [70] | * |  |  |  | * |  | * |  |  | NS |  |  | * |  |  | ** |  |  | * |  | 7 |
| Suren P/2013 [77] | * |  |  |  | * |  | * |  |  | ** |  |  |  | * |  | ** |  |  | * |  | 9 |
| Webb S/2007 [80] | * |  |  |  | * |  | * |  |  | ** |  |  | NS |  |  | ** |  |  | NS |  | 7 |

*, Acceptable; NS, not stated
