# Supplementary material for: The early life growth of head circumference, weight, and height in infants with autism spectrum disorders: a systematic review
Source: BMC Pediatr. 2023 Dec 8;23:619. doi: 10.1186/s12887-023-04445-9 (PMC10704616; doi:10.1186/s12887-023-04445-9)
Supplement: Supplementary file 3 — Supplementary Material 3 [file 12887_2023_4445_MOESM3_ESM.docx]

**Supplementary Table 4.** Newcastle-Ottawa scale for assessment of quality of 17 included case-control studies evaluating the early life growth of head circumference, weight, and height in infants with autism spectrum disorders.

| Study ID | Quality assessment criteria | | | | | | | | | | | | Overall Quality Score (Maximum = 9) |
| --- | --- | --- | --- | --- | --- | --- | --- | --- | --- | --- | --- | --- | --- |
|  | **Selection** | | | | | **Comparability** | | **Outcomes** | | | | |  |
|  | Is the case definition adequate? | Representativeness of the cases (how were cases selected) | Selection of Controls | | Definition of Controls | Comparability of cases and controls on the basis of the design or analysis | | Ascertainment of exposure | | | Same method of ascertainment for cases and controls | Non-Response rate (dropouts) |  |
|  | Yes, with independent validation | Consecutive or obviously representative series of cases | Community controls | Hospital controls | Healthy children | Study controls for the most important factor | Study controls other confounders | Secure record (e.g. surgical record/research records) | Structured interview where interviewer blind to case/control status | Interviewer not blinded to case/control status | yes | Same rate for both groups |  |
| Chawarska K/ 2011 [26] | * | * | * |  | * | NS | NS | * |  |  | * | * | 7 |
| Constantino J/ 2009 [65] | * | * | * |  | * |  | * | * |  |  | * | * | 8 |
| Daniel J/ 2014 [8] | * | * | * |  | * | * |  | * |  |  | * | * | 8 |
| Dementieva Y/ 2004 [20] | * | * | * |  | * | * |  | * |  |  | NS | * | 7 |
| Fukumoto A/2011 [69] | * | * | * |  | * |  | * | * |  |  | * | * | 8 |
| Grandgeorge M/2013 [9] | * | * | * |  | * |  | * |  | * |  | * | * | 8 |
| Hazlett H/2005 [71] | * | * | * |  | * |  | * | * |  |  | * | NS | 7 |
| Kral T/2018  [72] | * | * | * |  | * |  | * | * |  |  | * | * | 8 |
| Marz K/  2007 [21] | * | * | * |  | * | NS | NS |  | * |  | * | * | 7 |
| McKeague l/2015 [73] | * | * | * |  | * |  | * |  | * |  | * | * | 8 |
| Mraz K/  2009 [74] | * | * | * |  | * | * | NS |  | * |  | * | * | 8 |
| Muratori F/2012 [75] | * | * | * |  | * |  | * | * | * |  | * | * | 8 |
| Raghavan R/ 2018 [44] | * | * | * |  | * |  | * | * |  |  | * | * | 8 |
| Rommelse N/2011 [76] | * | * | * |  | * |  | * |  | * |  | * | * | 8 |
| Schrieken M/2013 [10] | * | * | * |  | NS |  | * | * |  |  | * | * | 7 |
| Torrey F/2004 [78] | * | * | * |  | NS |  | * | * |  |  | * | * | 7 |
| Van Daalen E/2007 [79] | * | * | * |  | NS |  | * | * |  |  | * | * | 7 |

*, Acceptable; NS, not stated
