# Supplementary material for: The early life growth of head circumference, weight, and height in infants with autism spectrum disorders: a systematic review
Source: BMC Pediatr. 2023 Dec 8;23:619. doi: 10.1186/s12887-023-04445-9 (PMC10704616; doi:10.1186/s12887-023-04445-9)
Supplement: Supplementary file 4 — Supplementary Material 4 [file 12887_2023_4445_MOESM4_ESM.docx]

**Supplementary Table 2.** The early life growth of head circumference, weight, and height in infants with autism spectrum disorders: A systematic review: Method of the database search strategy using PubMed, Scopus, ScienceDirect, Google Scholar, and Web of Sciences

| **Database (Search**  **conducted up to**  **February, 2022)** | **Search terms^a^** | **Number of studies searched** |
| --- | --- | --- |
| PubMed | ((((((((weight[Title/Abstract]) OR (height[Title/Abstract])) OR (length[Title/Abstract])) OR (head circumference[Title/Abstract])) OR (growth[Title/Abstract])) OR (nutritional status[Title/Abstract])) AND (((early life[Title/Abstract]) OR (first year of life[Title/Abstract])) OR (early growth[Title/Abstract]))) AND ((((autism spectrum disorders[Title/Abstract]) OR (ASD[Title/Abstract])) OR (autism[Title/Abstract])) OR (autistic[Title/Abstract]))) AND (((children[Title/Abstract]) OR (infants[Title/Abstract])) OR (infancy[Title/Abstract])) | 79 |
| SCOPUS | ( TITLE-ABS-KEY ( "weight" OR "height" OR "length" OR "head circumference" OR "growth" OR "nutritional status" ) ) AND ( TITLE-ABS-KEY ( "early life" OR "first year of life" OR "early growth" ) ) AND ( TITLE-ABS-KEY ( "autism spectrum disorders" OR "ASD" OR "autism" OR "autistic" ) ) AND ( TITLE-ABS-KEY ( "children" OR "infants" OR "infancy" ) ) AND ( LIMIT-TO ( LANGUAGE , "English" ) ) AND ( LIMIT-TO ( DOCTYPE , "ar" ) ) AND ( LIMIT-TO ( SUBJAREA , "MEDI" ) ) | 93 |
| Google Scholar | allintitle: "weight" OR "height" OR "length" OR "head circumference" OR "growth" OR "nutritional status” OR “early life" OR "first year of life" OR "early growth” AND "autistic" OR “autism” | 601 |
| ScienceDirect | Title, abstract, keywords: " early life weight" OR " early life height " OR " early life head" OR " early life nutritional status" AND " autism" | 51 |
| Total |  | 824 |

^a^Searches were limited to original articles, and studies published in the English language using the appropriate filters and/or search terms depending on the database.
